# Association between serum polyunsaturated fatty acids and bone mineral density in US adults: NHANES 2011-2014

**DOI:** 10.3389/fendo.2023.1266329

**Published:** 2023-11-16

**Authors:** Hao Liang, Chuang Xiong, Yuangang Luo, Jun Zhang, Yanran Huang, Runhan Zhao, Nian Zhou, Zenghui Zhao, Xiaoji Luo

**Affiliations:** ^1^ Department of Orthopedic Surgery, The First Affiliated Hospital of Chongqing Medical University, Chongqing, China; ^2^ Orthopedic Laboratory of Chongqing Medical University, Chongqing, China; ^3^ Department of Bone and Soft Tissue Oncology, Chongqing University Cancer Hospital, Chongqing, China; ^4^ Department of Orthopedics, Qianjiang Central Hospital of Chongqing, Qianjiang, Chongqing, China

**Keywords:** polyunsaturated fatty acids, docosapentaenoic acid (DPA), eicosadienoic acid (EDA), bone mineral density, NHANES

## Abstract

**Objective:**

The purpose of this study was to investigate the association between serum polyunsaturated fatty acids (PUFAs) and bone mineral density (BMD).

**Methods:**

We performed a cross-sectional study based on data from the National Health and Nutrition Examination Survey (NHANES) 2011-2014. The weighted multiple linear regression model was utilized to determine the association between serum PUFAs and BMD. Further smoothed curve fitting and threshold effect analysis were conducted. Finally, we performed a subgroup analysis.

**Results:**

In total, 1979 participants aged 20-59 years were enrolled. After adjusting for all covariates, we found that serum docosapentaenoic acid (DPA) was positively associated with head BMD (β = 0.0015, 95% Cl: 0.0004, 0.0026, P = 0.008296) and lumbar spine BMD (β = 0.0005, 95% Cl: 0.0000, 0.0010, P = 0.036093), and serum eicosadienoic acid (EDA) was negatively associated with thoracic spine BMD (β = -0.0008, 95% Cl: -0.0016, -0.0000, P = 0.045355). Smoothed curve fitting revealed a nonlinear positive association between serum DPA and lumbar spine BMD. Threshold effect analysis indicated that the threshold of serum DPA was 81.4 µmol/L. Subgroup analysis revealed a positive correlation between serum DPA and head BMD in the subgroup aged 50-59 years (β = 0.0025, 95% Cl: 0.0002, 0.0049, P = 0.035249) and females (β = 0.0026, 95% Cl: 0.0008, 0.0044, P = 0.005005). There was a positive relationship between serum DPA and lumbar spine BMD in females (β = 0.0008, 95% Cl: 0.0001, 0.0015, P = 0.017900) and a negative association between serum EDA and thoracic spine BMD in the subgroup aged 30-39 years (β = -0.0016, 95% Cl: -0.0031, -0.0001, P = 0.041331), males (β = -0.0012, 95% Cl: -0.0023, -0.0001, P = 0.039364) and other races (β = -0.0021, 95% Cl: -0.0037, -0.0006, P = 0.008059).

**Conclusion:**

This study demonstrated a linear positive relationship between serum DPA and head BMD, a nonlinear positive association between serum DPA and lumbar spine BMD, and a linear negative correlation between serum EDA and thoracic spine BMD in US adults.

## Introduction

As a global public health issue, osteoporosis is defined as a degenerative skeletal disorder that manifests as the disruption of bone microstructure and reduced bone mass, resulting in decreased bone strength and higher fracture risk (1, 2). According to a report by the Surgeon General (US), approximately 10 million (M) Americans over the age of 50 years are affected by osteoporosis (3). In Europe, approximately 22M women and 5.5M men have been diagnosed with osteoporosis (4). Most importantly, osteoporosis-related fragility fractures can lead to poor quality of life, severe economic burden, and significantly elevated mortality, especially hip fractures (5, 6). Therefore, the importance of exploring factors associated with osteoporosis should be emphasized.

Both genetic and nongenetic factors strongly correlate with the development of osteoporosis (7). Diet and nutrients, as nongenetic factors, have attracted more attention due to their impact on osteoporosis (8, 9). Polyunsaturated fatty acids (PUFAs) consist of two subtypes, n-3 and n-6, and are essential fatty acids acquired mainly through fish and vegetable oils. After consumption, PUFAs can be transformed into a sequence of long-chain derivatives in the human body. Alpha-linolenic acid (ALA) can be metabolized into eicosapentaenoic acid (EPA) and docosahexaenoic acid (DHA), all of which are crucial components of n-3 PUFAs. Linoleic acid (LA) can be converted into arachidonic acid (AA), both of which are fundamental constituents of n-6 PUFAs (10, 11). The role of n-6 PUFAs and their metabolites including prostaglandins, leukotrienes, and thromboxanes has been linked to various physiological processes such as inflammation generation, and platelet activation, while the n-3 PUFAs have been proven to trigger opposing physiological effects (12, 13). Previous studies have shown that PUFAs are associated with various chronic diseases, including cardiovascular events (14), diabetes (15), depressive disorders (16), osteoarthritis (17, 18), and osteoporosis (19, 20).

Bone mineral density (BMD) scores are widely utilized to evaluate bone mass and diagnose osteoporosis. However, the research evidence for the relationship between dietary PUFAs and BMD remains equivocal. One cohort study found a negative association between dietary PUFA intake and femoral neck BMD in premenopausal women (21). A cross-sectional study demonstrated a positive correlation between dietary intake of PUFAs and total BMD among adults aged 20–59 years (22). While a randomized clinical trial found no correlation between the supplementation with n-3 PUFAs and BMD was observed in kidney transplant recipients (23). These contradictory findings warrant additional investigation. In addition to dietary information, biological samples, such as serum, plasma, or red blood cells (RBCs), can be utilized for PUFA assessment. Currently, only limited research has explored the connection between PUFAs derived from biological samples and BMD (24, 25). Therefore, this research aims to investigate the correlation between serum PUFAs and BMD among adults aged 20-59 years using the National Health and Nutrition Examination Survey (NHANES) 2011-2014.

## Methods

### Study population

**Figure 1:**
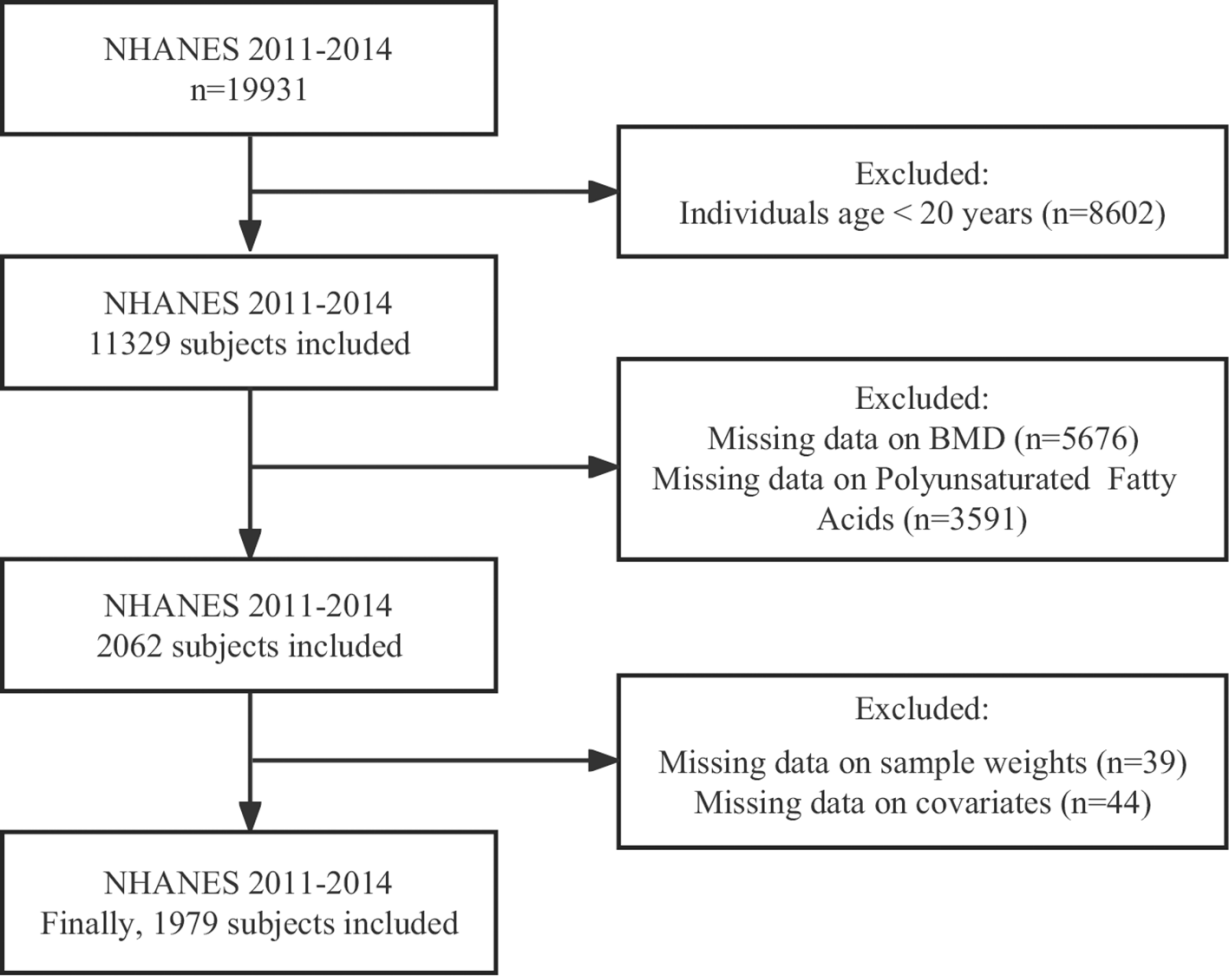
The selection flowchart of the participants.

NHANES is a health project that aims to investigate the health and nutrition status of the US population. The data of 19931 participants from the 2011-2014 cycle was utilized to evaluate the correlation between serum PUFAs and BMD, and to explore differential action of N-3 and N-6 PUFAs on bone. First, we eliminated subjects with ages less than 20 years (n=8602). Second, participants without complete information on serum PUFAs, head BMD, lumbar spine BMD, thoracic spine BMD, trunk BMD, and total BMD were excluded (n=9267). Third, we excluded participants missing information on sample weights (n=39) and covariates (n=44). Finally, we extracted 1979 participants in the study for final analysis. The inclusion and exclusion details of the study participants were shown in **Figure 1**. The NHANES study received approval from the NCHS Ethics Review Board and all participants provided written informed consent.

### Serum PUFAs measurements

Serum specimens were processed, stored, and shipped to the Division of Laboratory Sciences, National Center for Environmental Health, Centers for Disease Control and Prevention, Atlanta, GA for analysis. Fatty acids are detected using electron capture negative-ion mass spectrometry within 34 minutes. Finally, we selected 11 PUFAs for further analysis, including alpha-Linolenic acid (ALA, 18:3n-3), stearidonic acid (SDA, C18:4n-3), eicosapentaenoic acid (EPA, 20:5n-3), docosapentaenoic acid (DPA, 22:5n-3), docosahexaenoic acid (DHA, 22:6n-3), linoleic acid (LA, 18:2n-6), gamma-linolenic acid (GLA, 18:3n-6), eicosadienoic acid (EDA, 20:2n-6), homo-gamma-linolenic acid (HGLA, 20:3n-6), arachidonic acid (AA, 20:4n-6), docosapentaenoic acid (DPA, 22:5n-6).

### BMD measurements

Dual-energy X-ray absorptiometry (DXA) is a widely used technology for evaluating BMD owing to its rapidity, simplicity, and minimal radiation exposure (26). The DXA examinations based on QDR 4500A fan-beam densitometers (Hologic Inc) were administered by trained and certified radiology technologists and the whole body DXA scans provided the BMD data of the head, lumbar spine, thoracic spine, trunk and total body. Trunk BMD was defined as BMD measurements for trunk bone, including thoracic and lumbar spine, left and right ribs, and pelvis.

### Covariates

Confounding factors potentially associated with BMD were enrolled in this analysis. The demographic data included age, sex, race, and educational level. Body mass index (BMI) was defined as weight(kg) divided by the square of height(m2). Moderate recreational activities were obtained from the questionnaire (in a typical week do you do any moderate-intensity sports, fitness, or recreational activities that cause a small increase in breathing or heart rate such as brisk walking, bicycling, swimming, or volleyball for at least 10 minutes continuously) and we also included smoking data (smoked at least 100 cigarettes in life). Laboratory data included alkaline phosphatase, serum phosphorus, serum calcium, serum bilirubin, uric acid, total cholesterol, triglyceride, glycohemoglobin, and urine albumin creatinine ratio was collected from the fasting blood samples.

### Statistical analysis

The study analysis was performed by using EmpowerStats (http://www.empowerstats.com and R (4.2.3 version) software. Continuous variables were expressed as the mean ± standard deviation and categorical variables were expressed as numbers(n) and percentages (%). The weighted multiple linear regression model was utilized to determine the relationship between serum PUFAs and BMD. No covariates were adjusted in Model 1. Age, gender, and race were adjusted in Model 2. Age, gender, race, educational level, BMI, moderate recreational activities, smoked at least 100 cigarettes in life, alkaline phosphatase, serum phosphorus, serum calcium, serum bilirubin, uric acid, total cholesterol, triglyceride, glycohemoglobin, urine albumin creatinine ratio were adjusted in Model 3. Then, we conducted subgroup analysis by age, gender, and race. P < 0.05 was considered statistically significant. Further, we explored the association between serum PUFAs and BMD by using smoothed curve fitting and a weighted generalized additive model (GAM). The threshold effect of serum DPA on lumbar spine BMD was calculated using two-piece linear regression models.

## Results

### Baseline characteristics of participants

Of the 1979 study subjects, 1014(52%) were males and 965(48%) were females. Respectively, the average age of males and females was 38.85 ± 11.47 and 39.39 ± 11.69 years old. In terms of educational level, smoked at least 100 cigarettes in life, serum phosphorus, serum calcium, serum bilirubin, uric acid, triglyceride, urine albumin creatinine ratio, GLA, SDA, DPA, DHA, head BMD, thoracic Spine BMD, total BMD, we observed a significantly statistical difference between the two groups. There were no statistical differences in lumbar spine BMD and trunk BMD between the two groups. The weighted, detailed baseline information of the subjects was shown in **Table 1**.

**Table 1:** Baseline characteristics of participants.

Characteristic	Males, n = 1014 (52%)^1^	Females, n = 965 (48%)^1^	P Value^2^
Age (years)	38.85±11.47	39.39±11.69	0.361
Race/ethnicity, n (%)			0.181
Mexican American	130.00 (9.71%)	123.00 (9.38%)	
Other Hispanic	104.00 (7.30%)	104.00 (7.12%)	
Non-Hispanic White	424.00 (65.57%)	372.00 (62.78%)	
Non-Hispanic Black	164.00 (9.86%)	209.00 (12.28%)	
Other Race	192.00 (7.55%)	157.00 (8.44%)	
Educational level, n (%)			**0.004**
Less than 9th grade	56.00 (3.61%)	37.00 (2.53%)	
9-11th grade	144.00 (12.08%)	121.00 (10.90%)	
High school graduate/GED or equivalent	224.00 (23.02%)	171.00 (15.95%)	
Some college or AA degree	298.00 (30.08%)	335.00 (35.09%)	
College graduate or above	292.00 (31.21%)	301.00 (35.53%)	
Body mass index (kg/m2)	28.48±5.91	28.84±7.31	0.613
Smoked at least 100 cigarettes			**<0.001**
Yes	482.00 (47.40%)	307.00 (34.31%)	
No	532.00 (52.60%)	658.00 (65.69%)	
Moderate recreational activities			0.074
Yes	428.00 (42.76%)	453.00 (48.41%)	
No	586.00 (57.24%)	512.00 (51.59%)	
Alkaline phosphatase (IU/L)	63.38±17.35	62.74±20.67	0.151
Serum phosphorus (mmol/L)	1.17±0.17	1.23±0.17	**<0.001**
Serum calcium (mmol/L)	2.36±0.08	2.33±0.08	**<0.001**
Serum bilirubin (umol/L)	13.67±5.76	11.14±4.54	**<0.001**
Uric acid (umol/L)	364.07±69.69	276.70±63.06	**<0.001**
Total Cholesterol (mmol/L)	4.91±1.03	4.93±1.02	0.901
Triglyceride (mmol/L)	1.55±1.25	1.16±0.74	**<0.001**
Glycohemoglobin (%)	5.51±0.92	5.47±0.85	0.544
Urine albumin creatinine ratio (mg/g)	24.08±194.13	17.93±64.22	**<0.001**
Total n-6 PUFAs (umol/L)	4,791.80±1,210.66	4,677.43±1,033.95	0.257
LA (umol/L)	3,665.25±1,006.08	3,572.93±820.37	0.447
GLA (umol/L)	64.06±36.34	56.00±31.50	**<0.001**
EDA (umol/L)	23.16±9.41	22.41±7.89	0.413
HGLA (umol/L)	164.05±61.32	166.07±62.49	0.627
AA (umol/L)	854.74±241.84	838.85±260.28	0.133
DPA (umol/L)	20.53±8.39	21.17±9.59	0.655
Total n-3 PUFAs (umol/L)	352.25±159.99	348.24±141.49	0.516
ALA (umol/L)	91.99±59.71	83.87±41.69	0.139
SDA (umol/L)	4.14±3.60	3.57±2.77	**0.004**
EPA (umol/L)	61.32±44.31	58.75±45.14	0.262
DPA (umol/L)	54.88±21.66	47.75±18.09	**<0.001**
DHA (umol/L)	139.91±67.64	154.30±71.57	**<0.001**
n-6/n-3 (umol/L)	14.91±3.90	14.54±3.68	0.057
Head BMD (g/cm2)	2.12±0.32	2.29±0.38	**<0.001**
Lumbar Spine BMD (g/cm2)	1.03±0.15	1.03±0.15	0.387
Thoracic Spine BMD (g/cm2)	0.84±0.11	0.79±0.11	**<0.001**
Trunk BMD (g/cm2)	0.92±0.11	0.86±0.09	<0.001
Total BMD (g/cm2)	1.15±0.11	1.08±0.10	**<0.001**

^1^Mean±SD for continuous; n (unweighted) (%) for categorical.

^2^Wilcoxon rank-sum test for complex survey samples; chi-squared test with Rao & Scott's second-order correction.

Bold values represent statistical significance.

### Associations between serum PUFAS and BMD

We performed a weighted multiple linear regression model to investigate the relationship between serum PUFAs and BMD. After all the covariates were adjusted (model 3), we found that serum DPA was positively associated with head BMD (β = 0.0015, 95% Cl: 0.0004,0.0026, P = 0.008296) and lumbar spine BMD (β = 0.0005, 95% Cl: 0.0000,0.0010, P = 0.036093), serum EDA was negatively associated with thoracic spine BMD (β = -0.0008, 95% Cl: -0.0016,-0.0000, P = 0.045355). No relationship was observed between serum PUFAs and trunk BMD and total BMD, result details are shown in **Table 2**.

**Table 2:** Relationship between serum PUFAs and BMD.

	Head BMD		Lumbar spine BMD		Thoracic spine BMD		Trunk BMD		Total BMD	
	β(95%Cl)	P-value	β(95%Cl)	P-value	β(95%Cl)	P-value	β(95%Cl)	P-value	β(95%Cl)	P-value
LA	0.0000(-0.0000,0.0000)	0.643407	-0.0000(-0.0000,0.0000)	0.917583	0.0000 (-0.0000,0.0000)	0.217497	0.0000(-0.0000,0.0000)	0.532287	0.0000(-0.0000,0.0000)	0.401307
GLA	-0.0000(-0.0006,0.0005)	0.916053	0.0000(-0.0002,0.0003)	0.748661	-0.0000(-0.0002,0.0001)	0.623080	-0.0000(-0.0002,0.0002)	0.968531	-0.0000(-0.0002,0.0002)	0.973614
EDA	-0.0006(-0.0032,0.0020)	0.661861	-0.0003(-0.0014,0.0008)	0.625047	**-0.0008(-0.0016,-0.0000)**	**0.045355**	-0.0006(-0.0013,0.0002)	0.128612	-0.0003(-0.0010,0.0005)	0.453765
HGLA	0.0002(-0.0002,0.0005)	0.320140	0.0001(-0.0001,0.0002)	0.250818	0.0000 (-0.0001,0.0001)	0.951152	0.0000(-0.0001,0.0001)	0.418553	0.0001(-0.0000,0.0002)	0.077997
AA	0.0000(-0.0001,0.0001)	0.709653	0.0000(-0.0000,0.0001)	0.286111	-0.0000 (-0.0000,0.0000)	0.337952	-0.0000(-O0.0000,0.0000)	0.614450	-0.0000(-0.0000,0.0000)	0.296193
DPA	-0.0004(-0.0024,0.0016)	0.715433	0.0004(-0.0005,0.0012)	0.405885	-0.0003(-0.0009,0.0003)	0.371307	-0.0001(-0.0007,0.0005)	0.783197	-0.0002(-0.0007,0.0004)	0.510352
Total n-6 PUFAs	0.0000(-0.0000,0.0000)	0.549169	0.0000(-0.0000,0.0000)	0.735193	0.0000(-0.0000,0.0000)	0.430938	0.0000(-0.0000,0.0000)	0.648891	0.0000(-0.0000,0.0000)	0.578619
ALA	-0.0002(-0.0006,0.0003)	0.501371	-0.0001(-0.0003,0.0001)	0.364921	0.0000(-0.0001,0.0002)	0.802385	-0.0000(-0.0002,0.0001)	0.572231	-0.0000(-0.0001,0.0001)	0.962142
SDA	-0.0036(-0.0096,0.0025)	0.253160	-0.0022(-0.0048,0.0004)	0.097405	-0.0010(-0.0029,0.0008)	0.259009	-0.0010(-0.0028,0.0008)	0.268148	-0.0003(-0.0020,0.0014)	0.710131
EPA	0.0001(-0.0003,0.0004)	0.767557	-0.0000(-0.0002,0.0002)	0.975971	-0.0001(-0.0002,0.0000)	0.260300	-0.0000(-0.0001,0.0001)	0.589247	0.0000(-0.0001,0.0001)	0.778855
DPA	**0.0015 (0.0004,0.0026)**	**0.008296**	**0.0005(0.0000,0.0010)**	**0.036093**	-0.0001(-0.0005,0.0002)	0.411804	-0.0000(-0.0004,0.0003)	0.860290	0.0002(-0.0001,0.0005)	0.303189
DHA	-0.0001(-0.0004,0.0001)	0.334231	-0.0000(-0.0002,0.0001)	0.448581	-0.0000(-0.0001,0.0000)	0.412422	-0.0001(-0.0001,0.0000)	0.164544	-0.0001(-0.0001,0.0000)	0.123513
Total n-3 PUFAs	-0.0000(-0.0002,0.0001)	0.751520	-0.0000 (-0.0001,0.0000)	0.636003	-0.0000(-0.0001,0.0000)	0.364455	-0.0000(-0.0001,0.0000)	0.241699	-0.0000(-0.0001,0.0000)	0.529926
n-6/n-3	0.0006(-0.0038,0.0051)	0.783726	0.0003(-0.0015,0.0022)	0.727518	0.0007(-0.0006,0.0020)	0.303120	0.0006(-0.0006,0.0019)	0.327898	0.0004(-0.0009,0.0016)	0.546783

Age, gender, race, educational level, BMI, moderate recreational activities, smoked at least 100 cigarettes in life, alkaline phosphatase, serum phosphorus, serum calcium, serum bilirubin, uric acid, total cholesterol, triglyceride, glycohemoglobin, urine albumin creatinine ratio were adjusted in the weighted multiple linear regression model.

Bold values represent statistical significance.

### Smoothed curve fitting and threshold effect analysis

**Figure 2:**
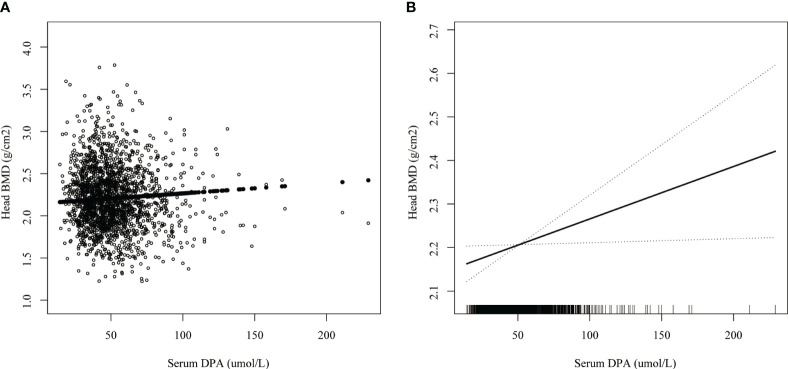
The association between serum DPA and head BMD. **(A)** each black point represents a sample. **(B)** the solid black line represents the smooth curve fit between variables. Dotted line bands represent the 95% confidence interval of the fit. Age, gender, race, educational level, BMI, moderate recreational activities, smoked at least 100 cigarettes in life, alkaline phosphatase, serum phosphorus, serum calcium, serum bilirubin, uric acid, total cholesterol, triglyceride, glycohemoglobin, urine albumin creatinine ratio were adjusted.

**Figure 3:**
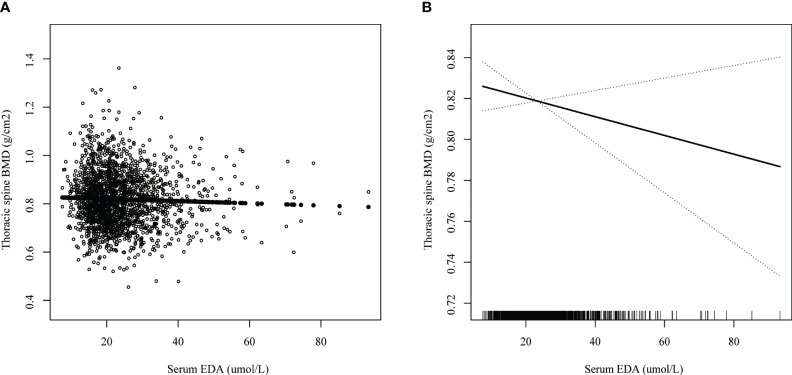
The association between serum EDA and thoracic spine BMD. **(A)** each black point represents a sample. **(B)** the solid black line represents the smooth curve fit between variables. Dotted line bands represent the 95% confidence interval of the fit. Age, gender, race, educational level, BMI, moderate recreational activities, smoked at least 100 cigarettes in life, alkaline phosphatase, serum phosphorus, serum calcium, serum bilirubin, uric acid, total cholesterol, triglyceride, glycohemoglobin, urine albumin creatinine ratio were adjusted.

**Figure 4:**
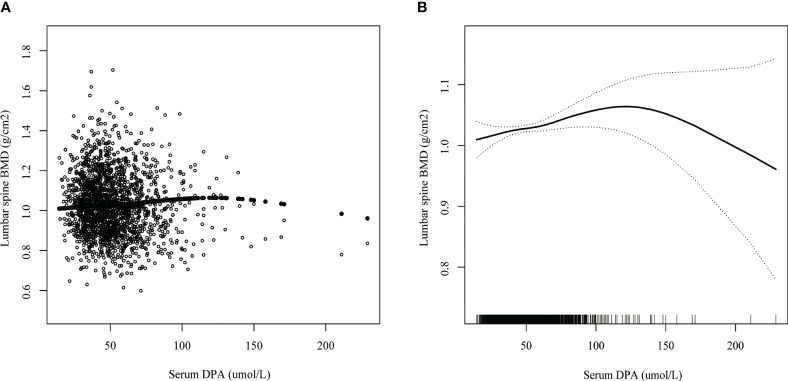
The association between serum DPA and lumbar spine BMD. **(A)** each black point represents a sample. **(B)** the solid black line represents the smooth curve fit between variables. Dotted line bands represent the 95% confidence interval of the fit. Age, gender, race, educational level, BMI, moderate recreational activities, smoked at least 100 cigarettes in life, alkaline phosphatase, serum phosphorus, serum calcium, serum bilirubin, uric acid, total cholesterol, triglyceride, glycohemoglobin, urine albumin creatinine ratio were adjusted.

Smoothed curve fitting revealed the linear association between serum DPA and head BMD (**Figure 2**), serum EDA, and thoracic spine BMD (**Figure 3**). A nonlinear association was found between serum DPA and lumbar BMD (**Figure 4**). Threshold effect analysis by using a two-piecewise linear regression model indicated the turning point of serum DPA was 81.4umol/L (**Table 3**). Serum DPA was positively associated with lumbar spine BMD (β = 0.0007, 95% Cl: 0.0001, 0.0012, P = 0.0208) when serum DPA <81.4umol/L. When serum DPA > 81.4umol/L, the relationship was not significant (β = 0.0001, 95% Cl: -0.0008, 0.0010, P = 0.8507).

**Table 3:** Threshold effect analysis of serum DPA (umol/L) and lumbar spine BMD(g/cm2) using two-piecewise linear regression model.

Lumbar spine BMD(g/cm2)	
Fitting by linear regression model	**0.0005 (0.0000, 0.0010) 0.0361**
Fitting by two-piecewise linear regression model	
Turn point of serum DPA (umol/L)	81.4
<81.4, effect 1	**0.0007 (0.0001, 0.0012) 0.0208**
>81.4,, effect 2	0.0001 (-0.0008, 0.0010) 0.8507
Log likelihood ratio test	0.305

Age, gender, race, educational level, BMI, moderate recreational activities, smoked at least 100 cigarettes in life, alkaline phosphatase, serum phosphorus, serum calcium, serum bilirubin, uric acid, total cholesterol, triglyceride, glycohemoglobin, urine albumin creatinine ratio were adjusted. Bold values represent statistical significance.

### Subgroup analysis

Furthermore, we conducted a subgroup analysis by age, gender, and race. In terms of serum DPA and head BMD, when stratified by age, a positive association between serum DPA and head BMD was observed in the subgroup aged 50-59 Years (β = 0.0025, 95% Cl: 0.0002,0.0049, P = 0.035249). When stratified by gender, we found that serum DPA was positively associated with head BMD in females (β = 0.0026, 95% Cl: 0.0008,0.0044, P = 0.005005), the result was shown in **Table 4**. In terms of serum DPA and lumbar spine BMD, we found a positive relationship in females (β = 0.0008, 95% Cl: 0.0001, 0.0015, P = 0.017900) when stratified by gender, the result was shown in **Table 5**. In terms of serum EDA and thoracic spine BMD, a negative association was found in the subgroup aged 30-39 Years (β = -0.0016, 95% Cl: -0.0031, -0.0001, P = 0.041331), males (β = -0.0012, 95% Cl: -0.0023, -0.0001, P = 0.039364) and other race (β = -0.0021, 95% Cl: -0.0037, -0.0006, P = 0.008059) when stratified by age, gender and race respectively, the result was shown in **Table 6**.

**Table 4:** Subgroup analysis of the relationship between serum DPA and head BMD.

	Model 1		Model 2		Model 3	
	β(95%Cl)	P-value	β(95%Cl)	P-value	β(95%Cl)	P-value
**Serum DPA**	-0.0005(-0.0013,0.0003)	0.210125	0.0002(-0.0006,0.0010)	0.649524	**0.0015(0.0004,0.0026**	**0.008296**
Stratified by age						
20-29 Years	-0.0016(-0.0034,0.0002)	0.087833	-0.0001(-0.0019,0.0018)	0.955135	0.0000(-0.0025,0.0025)	0.977817
30-39 Years	-0.0014(-0.0030.0.0002)	0.092035	0.0002(-0.0013.0.0018)	0.773166	0.0007(-0.0017,0.0031)	0.577333
40-49 Years	-0.0013(-0.0028,0.0002)	0.094098	0.0001(-0.0013,0.0016)	0.848408	0.0013(-0.0009,0.0035)	0.249357
50-59 Years	-0.0004(-0.0021,0.0012)	0.600252	-0.0001(-0.0017,0.0016)	0.948671	**0.0025(0.0002,0.0049)**	**0.035249**
Stratified by gender						
Male	0.0002(-0.0007,0.0011)	0.662386	0.0001(-0.0008,0.0011)	0.818739	0.0008(-0.0006,0.0023)	0.243991
Female	0.0002(-0.0011,0.0015)	0.754838	0.0002(-0.0012,0.0016)	0.784873	**0.0026(0.0008,0.0044)**	**0.005005**
Stratified by race						
Mexican American	-0.0004(-0.0024,0.0016)	0.703844	0.0010(-0.0012,0.0031)	0.366396	0.0016(-0.0014,0.0046)	0.285512
Other Hispanic	-0.0002(-0.0027,0.0024)	0.902879	0.0007(-0.0019,0.0034)	0.586048	0.0037(-0.0002,0.0075)	0.061983
Non-Hispanic white	0.0001(-0.0011,0.0012)	0.921793	0.0003(-0.0009,0.0015)	0.624622	0.0014(-0.0004,0.0032)	0.128920
Non-Hispanic black	-0.0023(-0.0050,0.0004)	0.093270	-0.0017(-0.0044,0.0010)	0.217913	0.0017(-0.0020,0.0054)	0.362165
Other race	-0.0005(-0.0020,0.0011)	0.557601	-0.0009(-0.0024,0.0007)	0.269804	0.0014(-0.0007,0.0036)	0.190573

Model 1: no covariates were adjusted.

Model 2: age, gender, and race were adjusted.

Model 3: age, gender, race, educational level, BMI, moderate recreational activities, smoked at least 100 cigarettes in life, alkaline phosphatase, serum phosphorus, serum calcium, serum bilirubin, uric acid, total cholesterol, triglyceride, glycohemoglobin, urine albumin creatinine ratio were adjusted. Bold values represent statistical significance.

**Table 5:** Subgroup analysis of the relationship between serum DPA and lumbar spine BMD.

	Model 1		Model 2		Model 3	
	β(95%Cl)	P-value	β(95%Cl)	P-value	β(95%Cl)	P-value
**Serum DPA**	-0.0004 (-0.0008, -0.0001)	0.007858	-0.0002 (-0.0005, 0.0002)	0.379742	**0.0005 (0.0000, 0.0010)**	**0.036093**
Stratified by age						
20-29 Years	0.0001 (-0.0007, 0.0010)	0.759573	0.0003 (-0.0006, 0.0012)	0.490057	-0.0001 (-0.0013, 0.0010)	0.834775
30-39 Years	-0.0003 (-0.0009, 0.0004)	0.441149	-0.0001 (-0.0007, 0.0006)	0.866197	0.0006 (-0.0004, 0.0017)	0.222370
40-49 Years	-0.0009 (-0.0015, -0.0002)	0.008880	-0.0005 (-0.0012, 0.0001)	0.125960	0.0003 (-0.0007, 0.0012)	0.580044
50-59 Years	-0.0000 (-0.0006, 0.0006)	0.922797	-0.0001 (-0.0007, 0.0005)	0.796055	0.0007 (-0.0002, 0.0016)	0.119122
Stratified by gender						
Male	-0.0005 (-0.0009, -0.0000)	0.036530	-0.0003 (-0.0007, 0.0001)	0.197322	0.0003 (-0.0003, 0.0010)	0.324876
Female	-0.0004 (-0.0009, 0.0001)	0.160154	0.0001 (-0.0004, 0.0007)	0.663739	**0.0008 (0.0001, 0.0015)**	**0.017900**
Stratified by race						
Mexican American	-0.0003 (-0.0011, 0.0005)	0.437910	-0.0001 (-0.0009, 0.0007)	0.853077	0.0001 (-0.0011, 0.0012)	0.882147
Other Hispanic	0.0003 (-0.0007, 0.0014)	0.527626	0.0010 (-0.0002, 0.0021)	0.094548	0.0010 (-0.0006, 0.0027)	0.212801
Non-Hispanic white	-0.0003 (-0.0008, 0.0002)	0.203654	-0.0002 (-0.0007, 0.0004)	0.549060	0.0005 (-0.0003, 0.0013)	0.188309
Non-Hispanic black	-0.0011 (-0.0022, 0.0000)	0.058988	-0.0009 (-0.0021, 0.0003)	0.144914	0.0006 (-0.0010, 0.0022)	0.493524
Other race	-0.0006 (-0.0012, 0.0001)	0.090649	-0.0004 (-0.0011, 0.0003)	0.255681	0.0008 (-0.0001, 0.0017)	0.084806

Bold values represent statistical significance.

**Table 6:** Subgroup analysis of the relationship between serum EDA and thoracic spine BMD.

	Model 1		Model 2		Model 3	
	β(95%Cl)	P-value	β(95%Cl)	P-value	β(95%Cl)	P-value
**Serum EDA**	-0.0005 (-0.0010, 0.0001)	0.110023	-0.0005 (-0.0010, 0.0001)	0.082590	**-0.0008 (-0.0016, -0.0000)**	**0.045355**
Stratified by age						
20-29 Years	0.0005 (-0.0005, 0.0016)	0.326502	0.0010 (-0.0000, 0.0021)	0.055493	0.0004 (-0.0011, 0.0018)	0.619783
30-39 Years	-0.0004 (-0.0014, 0.0007)	0.516746	-0.0003 (-0.0014, 0.0007)	0.546388	**-0.0016 (-0.0031, -0.0001)**	**0.041331**
40-49 Years	-0.0003 (-0.0013, 0.0008)	0.582345	-0.0003 (-0.0013, 0.0007)	0.580130	-0.0003 (-0.0018, 0.0011)	0.663472
50-59 Years	-0.0020 (-0.0033, -0.0007)	0.003020	-0.0023 (-0.0035, -0.0010)	0.000356	-0.0019 (-0.0039, 0.0001)	0.058075
Stratified by gender						
Male	-0.0003 (-0.0010, 0.0004)	0.453229	-0.0004 (-0.0011, 0.0003)	0.263614	**-0.0012 (-0.0023, -0.0001)**	**0.039364**
Female	-0.0011 (-0.0019, -0.0002)	0.016808	-0.0008 (-0.0016, 0.0001)	0.078244	-0.0007 (-0.0018, 0.0004)	0.215719
Stratified by race						
Mexican American	0.0010 (-0.0001, 0.0020)	0.077203	0.0007 (-0.0004, 0.0017)	0.206954	-0.0002 (-0.0019, 0.0015)	0.782629
Other Hispanic	-0.0001 (-0.0016, 0.0014)	0.884382	-0.0003 (-0.0018, 0.0013)	0.730043	-0.0015 (-0.0038, 0.0007)	0.187988
Non-Hispanic white	-0.0002 (-0.0011, 0.0007)	0.674408	-0.0004 (-0.0013, 0.0005)	0.418354	-0.0007 (-0.0020, 0.0006)	0.292860
Non-Hispanic black	-0.0031 (-0.0052, -0.0010)	0.003744	-0.0030 (-0.0051, -0.0009)	0.005217	-0.0002 (-0.0030, 0.0026)	0.882039
Other race	-0.0015 (-0.0027, -0.0003)	0.012820	-0.0022 (-0.0034, -0.0010)	0.000402	**-0.0021 (-0.0037, -0.0006)**	**0.008059**

Bold values represent statistical significance.

## Discussion

For the first time, we utilized the NHANES database to evaluate the association between serum PUFAs and BMD. This study demonstrated a nonlinear positive association between serum DPA and lumbar spine BMD, a linear positive relationship between serum DPA and head BMD, and a linear negative correlation between serum EDA and thoracic spine BMD in US adults. Therefore, we speculated that serum n-3 PUFAs were beneficial for BMD, while n-6 PUFAs had the opposite effect.

Recent years have seen accumulating evidence suggesting potential associations between PUFAs and various human diseases (14–16), including those related to bone health (19, 20). Numerous clinical studies have delved into the relationship between dietary PUFAs and bone health. For instance, a cross-sectional study based on NHANES database demonstrated a positive correlation between total dietary intake of PUFAs and total BMD among adults aged 20–59 years (22). A positive association was also discerned between the consumption of PUFAs intake and both total BMD and lumbar spine BMD (27). However, it should be noted that this association appeared to be limited to the specific demographic of older women without hormone therapy which limited the applicability of this conclusion to other populations. required verification. On the contrary, a longitudinal study demonstrated that an increase in dietary intake of PUFAs and monounsaturated fatty acids (MUFAs) was correlated with decreased femoral neck BMD in women aged 45–55 years of age (21). In addition, total dietary PUFAs intake increased the fracture risk in the older age group >65 years, according to Martínez-Ramírez et al. (28). However, a cohort research demonstrated no association between total dietary PUFAs consumption and hip fracture risk (29). These studies presented varied results regarding the effect of total dietary intake of PUFAs on BMD or fracture risk. The inconsistencies could potentially be attributed to specific factors such as age, gender, and the site of BMD measurement in the study population, which highlighted the need for further and comprehensive investigation. In addition, differences in the impact of dietary PUFAs subgroups on bone health should also be given due consideration.

In fact, the mechanisms by which n-3 PUFAs and n-6 PUFAs function within various body tissues, including bones, have been revealed to differ (12, 30). A pivotal factor is the direction of mesenchymal stem cell (MSC) differentiation, which steers either osteogenesis or adipogenesis. The peroxisome proliferator-activated receptor γ (PPARγ) has a crucial role in driving the differentiation of MSC into adipocytes, subsequently inhibiting osteogenesis (31). Existing studies have shown that n-6 PUFAs impede osteogenesis through the upregulation of PPARγ expression and downregulation of Runx2 expression (32), whereas n-3 PUFAs manifest converse effects (33). N-6 PUFAs are also known to trigger RANKL–RANK signaling, leading to osteoclastogenesis and bone loss (32, 34), a process which is suppressed by n-3 PUFAs (35, 36). Furthermore, n-6 PUFAs are found to elevate pro-inflammatory cytokine levels, promoting bone resorption (37, 38). The outcomes of these experimental findings propose that n-3 PUFAs exert beneficial influences on bone health, standing in contrast to the detrimental effects of n-6 PUFAs. Therefore, further assessment of the effects of different PUFA subclasses on human bone health is required.

A cross-sectional study utilizing the NHANES database showed that dietary supplementation with n-3 PUFAs (EPA, DHA, and SDA) was positively associated with lumbar spine BMD among adults >60 years of age (39). Correspondingly, another cross-sectional study reported a beneficial impact of dietary n-3 PUFAs consumption on lumbar spine BMD in postmenopausal women (40). Beyond its impact on lumbar spine BMD, dietary intake of n-3 PUFAs was also found to enhance hip BMD in the female population aged 19-25 years (41). In addition, dietary n-3 PUFAs intake reduced the levels of biological markers of bone resorption, suggesting that n-3 PUFAs reduced bone loss potentially by inhibiting osteoclast activity (42). While the majority of studies, encompassing the aforementioned ones, endorsed the beneficial effects of dietary n-3 PUFAs on bone health, aligning with prior experimental results, there still existed certain studies that reached inconsistent conclusions. Within the context of a randomized clinical trial, no correlation between n-3 PUFAs supplementation and BMD was observed amongst kidney transplant recipients (23). Furthermore, a comprehensive meta-analysis also suggested that supplementation with dietary n-3 PUFAs had no positive impact on BMD (43), a conclusion that deviated from the results of experimental findings. We proposed that two principal factors contributed to this issue. First, the majority of studies have concentrated on establishing an association between dietary PUFAs consumption and BMD or fracture risk. Nonetheless, data on dietary PUFAs was predominantly obtained from food-frequency questionnaires, which may result in an inaccurate assessment of PUFAs intake. Second, dietary intake of PUFAs did not fully align with bioavailable PUFAs, a factor that could be influenced by the digestion and absorption process. Our hypothesis has garnered support from various studies. For instance, one study highlighted that dietary PUFAs intake was not correlated with serum levels of PUFAs (44). Another study demonstrated that the dietary intake of ALA did not impact its plasma levels, with the connection between dietary consumption and plasma concentration only proving significant for LA, AA, EPA, and DHA (45). Therefore, we advocate the use of biological specimens such as serum, plasma, or red blood cells (RBCs) for a more accurate assessment of PUFAs as opposed to relying solely on dietary information.

However, only a limited number of studies have investigated the association between PUFA levels in biological samples and BMD. A cross-sectional study involving 301 Spanish postmenopausal women demonstrated a positive correlation between the plasma concentration of n-3 PUFAs (inclusive of ALA, EPA, and DHA) and BMD in the spine and neck of the femur (24). A significantly positive correlation between the serum concentration of n-3 PUFAs and femur BMD was observed exclusively within the group having a low n-6:n-3 ratio in another study (25), suggesting that high serum concentrations of n-6 PUFAs potentially impeded the bone health-promoting effects of n-3 PUFAs. This study also showed that the serum concentration of ALA, a class of n-3 PUFAs, was negatively correlated with creatinine-corrected urinary deoxypyridinoline, suggesting that n-3 PUFAs may promote BMD by inhibiting bone resorption. Moreover, PUFA levels from the biological samples were also associated with fracture risk. In a cohort study including 1438 participants, there was a decrease in fracture risk with increased plasma levels of n-3 PUFAs and EPA, while n-6 PUFAs and AA manifested the contrary effects (46). A nested case−control study found that elevated levels of total n-3 PUFAs, ALA, and EPA derived from RBCs, along with a high n-6:n-3 ratio, were associated with a decreased risk of hip fracture (47). These studies indicated that high level of n-3 PUFAs from biological samples were beneficial for promoting BMD or mitigated fracture risk, whereas n-6 PUFAs exerted an opposing effect. Although the conclusions drawn from these studies aligned well with the outcomes of prior experimental results, certain limitations, such as the sample size, the diversity in the types of PUFAs assessed, and the limited number of sites for BMD measurement, needed to be duly acknowledged.

It was worth noting that BMD in various regions had varying clinical significance. Low BMD in the spine might be linked to fragility fractures. Moreover, low head BMD could be connected to hearing impairments (48) and malocclusion in adolescents (49). Therefore, to improve the comprehensiveness and reliability of the study, we included 11 serum PUFAs and 5 sites of BMD to investigate their associations using the NHANES large sample data (n=1979). First, our study revealed a nonlinear positive association between serum DPA and lumbar spine BMD, a relationship that remained consistent solely within the female subgroup but was absent in the male subgroup. Moreover, threshold effect analysis indicated that when serum DPA > 81.4umol/L, the positive relationship was no more significant. Regarding the threshold and nonlinear relationship, previous research proposed that excess dietary intake of n-3 PUFAs beyond the threshold level conferred no additional benefits to bone health (23). In a related animal study, increased consumption of DHA, a type of n-3 PUFA, was shown to benefit BMD, bone mineral content, and peak bone mass. However, no additional benefits were not observed in the group with higher intake of DHA (50). Therefore, we postulated that a threshold might exist for the promotion of lumbar spine BMD by some of the serum N-3 PUFAs, such as DPA. However, this hypothesis warranted further validation through additional clinical studies. Second, our investigation discerned a linear and positive relationship between serum DPA and head BMD, a trend that was consistent within the female subgroup, though not apparent in the male counterpart. A meta-analysis demonstrated that n-3 PUFA supplementation had a better favorable impact on BMD in females (43). An animal study reported that female offspring of mice supplemented with n-3 PUFA had better bone health than male offspring (51). These studies suggested that there was a potential sex differences in the promotion of BMD by n-3 PUFAs, which explained why the positive association of DPA with head BMD and lumbar spine BMD was only significant in the female subgroup in this study. In addition, the positive trend between serum DPA and head BMD was significant in the subgroup aged 50-59 years. BMD decreased with age, potentially highlighting the importance of DPA in promoting BMD among participants aged 50-59 years. Regarding the subtle differences in the effects of serum DPA on head BMD and lumbar spine BMD, we speculated that they are related to the different morphology and activity of osteoclasts, osteogenic capacity of bone marrow stromal cells at the different sites (52–54).Third, our research demonstrated that serum EDA levels had a linear and negative correlation with thoracic spine BMD, which remained consistent within the subgroup aged 30-39 years, males and other race. However, we needed further evidence from large clinical studies to support this subgroup association.

This study had some advantages. First, the research data were extracted from the NHANES database, which ensured the accuracy and representativeness of the data. Second, covariates potentially associated with BMD were adjusted to improve the reliability of this study. However, limitations cannot be ignored. First, it is important to note that this study was a cross-sectional study, which precluded drawing causal inferences regarding serum PUFAs and BMD. Second, this study focused on participants 20-59 years of age, and the conclusions cannot be directly extrapolated to older populations or adolescents. Third, the levels of PUFAs from biological samples were more difficult to obtain compared to food frequency questionnaires and had higher economic costs. Fourth, the relationship between serum PUFAs and BMD was weak. Finally, there is a lack of experimental validation in this study.

In the future, we will conduct further studies focusing on the following aspects. First, we will design clinical randomized controlled trials to investigate the causal association between serum PUFAs and BMD. Second, we aim to expand the study to include elderly individuals who are at high risk for osteoporosis and adolescents. Third, we will investigate the relationship between dietary PUFA intake and serum PUFA levels and ascertain the factors that have an impact on this relationship. Fourth, we will experimentally validate the effects of PUFAs on osteogenesis markers, adipogenesis markers, and inflammatory markers (TNF-α, IL-1β, IL-6, and COX-2).

## Conclusions

In conclusion, this study demonstrated a linear positive relationship between serum DPA and head BMD, a nonlinear positive association between serum DPA and lumbar spine BMD, and a linear negative correlation between serum EDA and thoracic spine BMD in US adults aged 20-59 years.

## Data availability statement

Publicly available datasets were analyzed in this study. This data can be found here: https://www.cdc.gov/nchs/nhanes/index.htm.

## Ethics statement

The studies involving humans were approved by NCHS Research Ethics Review Board (ERB). The studies were conducted in accordance with the local legislation and institutional requirements. The participants provided their written informed consent to participate in this study.

## Author contributions

HL: Formal Analysis, Methodology, Writing – original draft, Writing – review & editing. CX: Formal Analysis, Methodology, Writing – original draft, Writing – review & editing. YL: Methodology, Software, Writing – review & editing. JZ: Methodology, Software, Supervision, Writing – review & editing. YH: Methodology, Software, Writing – review & editing. RZ: Methodology, Software, Writing – review & editing. NZ: Supervision, Writing – review & editing. ZZ: Supervision, Writing – review & editing. XL: Funding acquisition, Supervision, Validation, Writing – review & editing.
